# A Case of Multiple Omphalomesenteric Duct Anomalies in a 15‐Year‐Old Female

**DOI:** 10.1002/ccr3.73930

**Published:** 2026-11-11

**Authors:** Tayyab Riaz, Mohammad Abdullah Hameed, Ritik Singh

**Affiliations:** ^1^ Abwa Hospital & Research Center Faisalabad Pakistan; ^2^ CMH Lahore Medical College and Institute of Dentistry Lahore Pakistan; ^3^ Danylo Halytsky Lviv National Medical University Lviv Ukraine

**Keywords:** fibrous band, Meckel's diverticulum, OMD anomalies, omphalomesenteric cyst, omphalomesenteric duct, umbilical discharge, umbilical polyp, umbilical sinus

## Abstract

Adolescents with symptomatic abnormalities of the omphalomesenteric duct are quite uncommon. We describe a 15‐year‐old who has an umbilical polyp, cyst, sinus, Meckel's diverticulum, and a fibrous band linking them. This case emphasizes the importance of comprehensive surgical investigation and total excision in atypical umbilical complaints, especially beyond infancy.

## 1 Introduction

The omphalomesenteric duct (OMD), also referred to as the vitellointestinal duct (VID) or the vitelline duct, is an embryological structure that serves to connect the midgut to the yolk sac in early fetal development. It plays a transient nutritional role before spontaneously obliterating during the 5th–10th week of gestation, ultimately separating from the intestine. In about 2% of individuals, partial or complete failure of OMD involution can result in a spectrum of congenital anomalies [1]. The most frequently occurring anomaly is Meckel's diverticulum (MD), while others include vitelline cord, OMD cyst, fistula, umbilical polyp, and fibrotic band—each anomaly reflecting persistence of the duct at a different segment or degree of closure failure [1, 2, 3].

The clinical presentation varies depending on the specific type of anomaly present. Secondary complications may also develop including umbilical discharge, acute abdomen, intestinal obstruction, gastrointestinal bleeding, or intussusception [1]. OMD remnants typically go unnoticed unless such complications arise, which usually occurs within the pediatric population, particularly in the first 4 years of life. As the individual progresses to adolescence or adulthood, the incidence of these complications and hence the detection of the OMD remnants becomes exceedingly uncommon [3, 4]. Additionally, while isolated anomalies have been relatively widely reported, coexistence of multiple OMD remnants in a single individual is exceptionally rare and sparsely documented in existing literature.

Prompt recognition, clinical diagnosis and treatment of these congenital anomalies is important to prevent complications. Herein, we report a rare case of a 15‐year‐old girl with a set of coexisting OMD anomalies which, to the best of our knowledge, have not been documented in previous literature.

## 2 Case History/Examination

A 15‐year‐old girl presented to the outpatient department with a primary complaint of umbilical discharge for 2 weeks. The discharge was whitish in color, continuous, and not foul‐smelling. She also reported localized umbilical pain for 3 weeks, which subsided following the onset of discharge. There was no associated fever, vomiting, altered bowel habits, gastrointestinal bleeding, or weight loss. She had no prior history of similar complaints, abdominal surgery, or significant medical treatment. Menstrual history was normal, and there was no history of tuberculosis contact.

On examination, the patient was clinically stable with normal vital signs. Local examination revealed a papillomatous growth at the umbilicus, consistent with an umbilical polyp (see Figure 1). There was also an active whitish umbilical discharge. The surrounding skin showed no erythema, induration, or signs of cellulitis. The abdomen was soft and non‐tender, with no palpable masses or organomegaly. Systemic examination was unremarkable.

**FIGURE 1:**
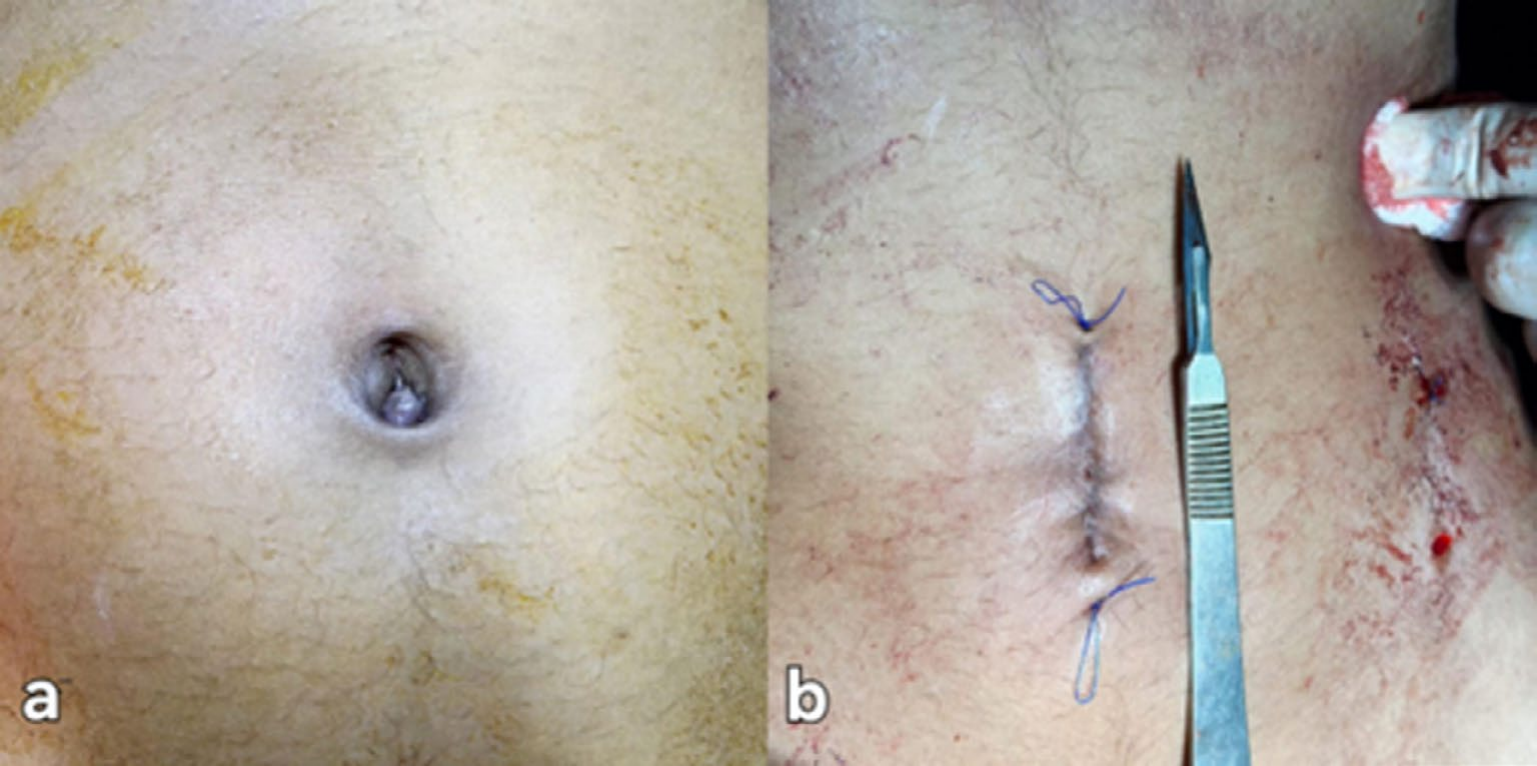
Umbilical lesion before and after surgery. (a) Papillomatous umbilical polyp. (b) Postoperative appearance following omphalectomy and excision of the lesion.

## 3 Differential Diagnosis, Investigations, and Treatment

Ultrasonography of the abdomen demonstrated a well‐defined, ovoid cystic lesion measuring 27 × 17 mm in the anterior abdominal wall, with a tract extending to the umbilicus, suggestive of an OMD anomaly (see Figure 2). Sinogram, computed tomography (CT) scan, and magnetic resonance imaging (MRI) were considered as alternative diagnostic modalities; however, the primary surgeon determined that diagnostic laparoscopy offered the optimal approach for directly visualizing the pathology and facilitating immediate subsequent intervention under the same anesthesia, thereby providing economic benefits to the patient. No communication with the urinary bladder or bowel was observed. Routine laboratory investigations were within normal limits.

**FIGURE 2:**
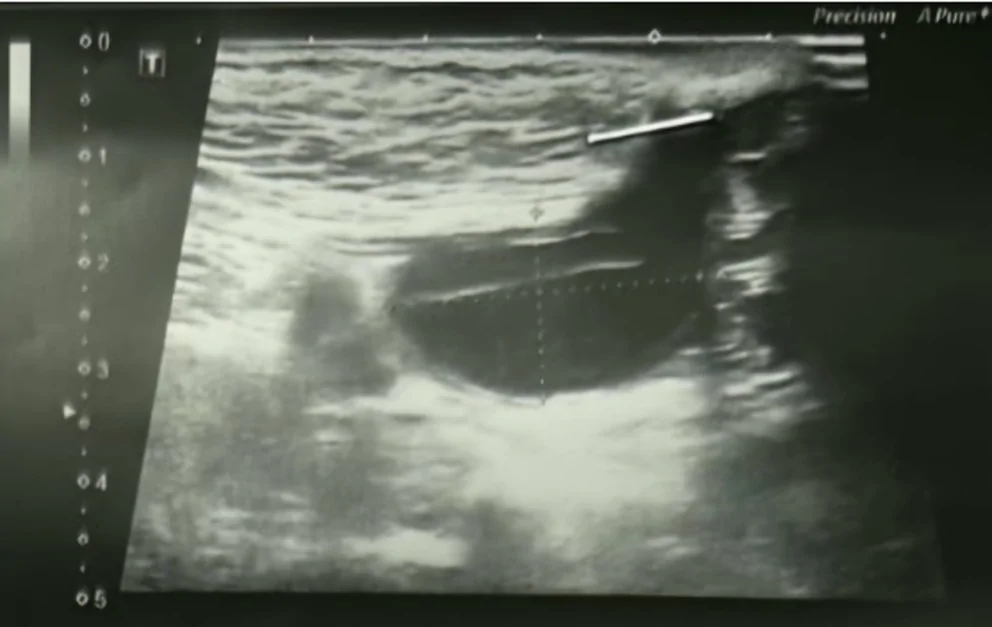
Ultrasonography of the umbilical region showing a well‐defined tract extending from the umbilicus to the cystic lesion.

Diagnostic laparoscopy revealed a cystic OMD remnant in continuity with the umbilicus. This was most likely an omphalomesenteric cyst that had remained clinically silent and subsequently developed secondary communication with the umbilicus, resulting in sinus formation and discharge. An MD arising from the ileum was noted, measuring approximately 3 in. in length and located 2 ft proximal to the ileocecal junction. The MD was connected to the cyst by a fibrous band, confirming persistent OMD remnants (see Figure 3).

**FIGURE 3:**
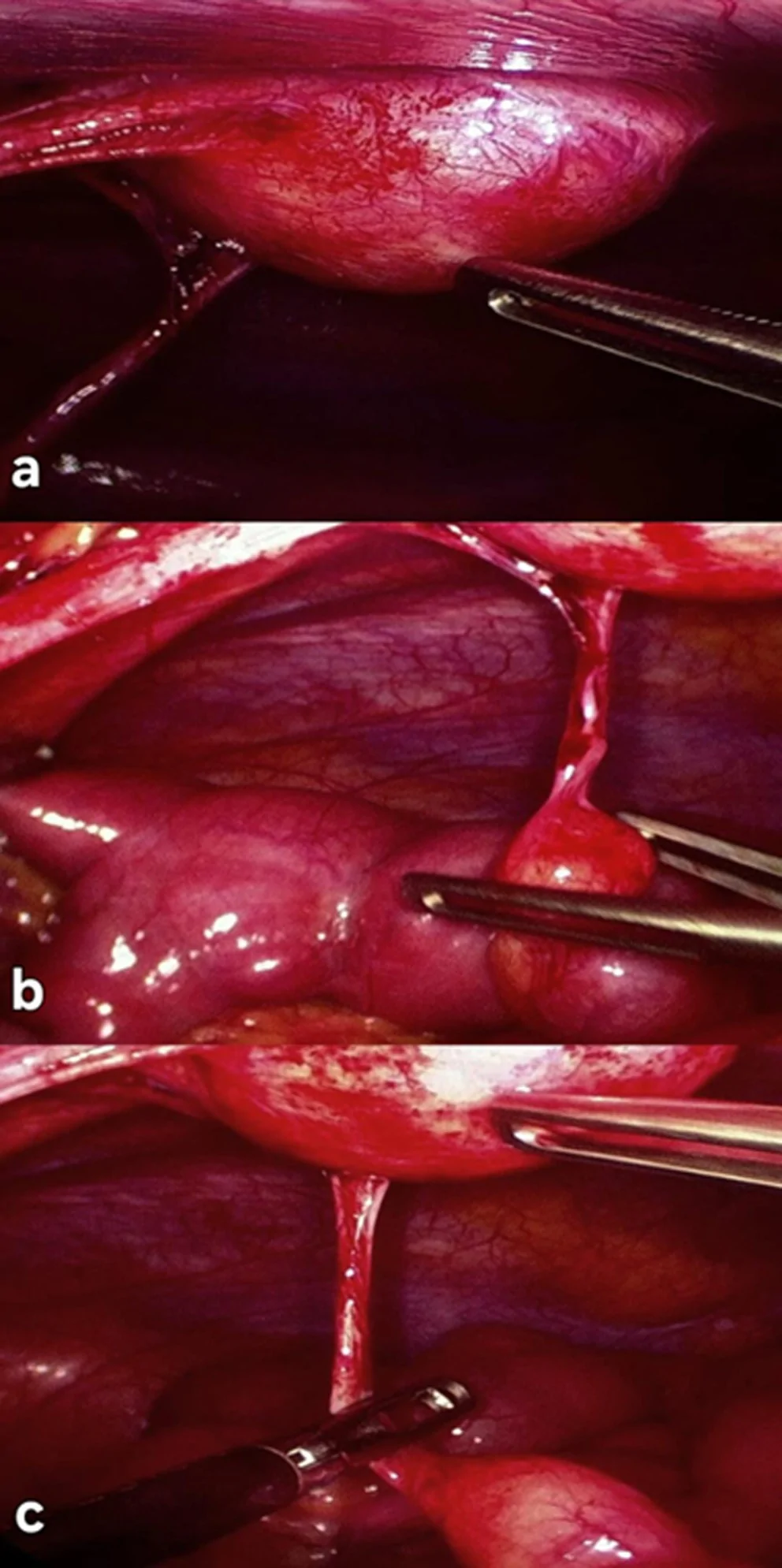
Diagnostic laparoscopy findings. (a) Laparoscopic view of the omphalomesenteric cyst. (b) MD visualized intra‐abdominally. (c) MD connected to the cyst via a fibrous band, confirming persistent OMD remnants.

An elliptical incision of approximately 5 cm was made around the umbilicus and omphalectomy with complete excision of the umbilical polyp, sinus tract, and cyst was performed. The MD was delivered through the incision, followed by segmental ileal resection and end‐to‐end ileo‐ileal anastomosis. On opening the cyst cavity, purulent material was encountered. An unusual finding of a small leaf fragment was noted within the cyst cavity, likely introduced through the persistent umbilical sinus (see Figure 4).

**FIGURE 4:**
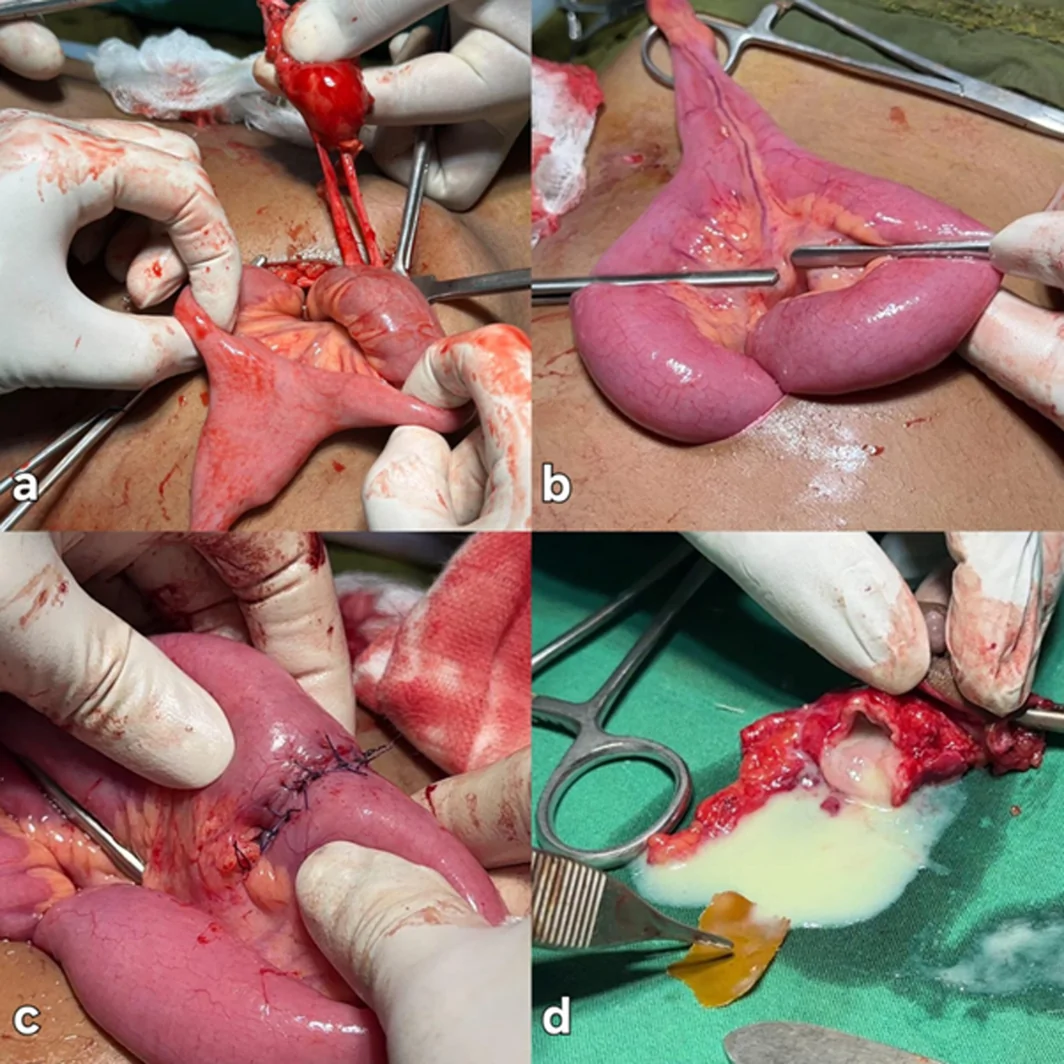
Intraoperative findings and surgical procedure. (a) Omphalectomy with excision of the cyst through a 5 cm elliptical umbilical incision. (b) Meckel's diverticulum delivered through the incision. (c) Segmental ileal resection and end‐to‐end ileo‐ileal anastomosis following excision of the diverticulum. (d) Opened cystic cavity showing purulent contents and a small leaf that was retrieved from it.

Histopathological examination, performed using hematoxylin and eosin (H&E) staining of the excised umbilical tissue and MD, revealed moderate, nonspecific inflammation, and hyperplasia of mucosal lymphoid tissue, with no evidence of granulomatous inflammation or malignancy, supporting the diagnosis of an umbilical polyp.

## 4 Conclusion and Results (Outcome and Follow‐Up)

The postoperative period was uneventful. The patient was discharged in stable condition and followed up at 7 days, 2 weeks, and 1 month, during which she remained asymptomatic with satisfactory wound healing and no recurrence of symptoms. This case highlights the coexistence of multiple OMD anomalies in a 15‐year‐old girl presenting with umbilical discharge. A high index of suspicion, appropriate imaging, and timely surgical management are crucial for optimal outcomes. Awareness of such combinations is important for surgeons when evaluating atypical umbilical complaints beyond infancy, particularly in adolescents.

## 5 Discussion

OMD anomalies represent a continuum of embryological remnants, the most common being MD. Other anomalies such as umbilical sinus, omphalomesenteric cyst, fibrous bands, and umbilical polyps are much less frequent [1, 2, 3]. Additionally, the presence of an omphalomesenteric cyst is rather rare in itself, and such cysts seldom become symptomatic—their combination with other anomalies scarcely being documented [5]. The coexistence of multiple OMD anomalies in a single patient is extremely rare, especially in adolescence, as most symptomatic cases present in infancy or early childhood [3, 4]. Although the incidence of these malformations is found to be equal among both genders, symptomatic OMD remnants are significantly more frequent in males [1, 3]. In this report, we describe a rare combination of OMD anomalies in a 15‐year‐old girl, including an umbilical polyp, an omphalomesenteric cyst that later formed an umbilical sinus, MD and a fibrous band connecting the cyst to the MD.

These different OMD anomalies represent a distinct segment or degree of closure failure [1, 2, 3]. An MD is encountered when the proximal duct persists. If the duct remains completely patent, an omphalomesenteric fistula develops between the umbilicus and the ileum; persistence of the distal segment results in a sinus tract, while patency of the intermediate segment with closure at both ends leads to formation of an omphalomesenteric cyst [6]. A relevant question is whether the constellation of anomalies observed in our patient reflects a single, total failure of OMD involution or several independent, segmental failures of closure occurring along the length of the duct. We favor the latter interpretation. A true total failure of involution i.e., a fully patent duct from ileum to umbilicus, would be expected to produce a single continuous fistulous tract with free enteric communication, which was not observed in this case. Instead, the four anatomically distinct components (umbilical polyp, sinus, cyst, and MD) were separated by regions of obliteration, with the MD and cyst joined only by a non‐patent fibrous cord rather than a patent lumen. The presence of this fibrous band is itself evidence of localized, incomplete closure at that segment rather than persistent patency, supporting a model of multifocal segmental failure along the duct rather than a single uniform developmental defect. This interpretation is consistent with the similar fibrous‐band configuration reported by Ioannidis et al. [6], suggesting that multifocal segmental persistence, while rare, may represent a recognizable subtype of complex OMD anomaly. In our case, we suspect that before the patient experienced any symptoms, an omphalomesenteric cyst was initially present which later developed a secondary communication with the umbilicus, after which the patient became symptomatic with umbilical discharge. This occurrence closely resembles the reported findings by Ioannidis et al. and Jonuzi et al., making our case one of the few reported instances of an omphalomesenteric cyst communicating with the umbilicus via a sinus tract [6, 7].

An umbilical OMD polyp arises when a portion of the OMD persists at the umbilicus without maintaining a patent connection to the bowel. The polyp is lined by intestinal mucosa, which can predispose it to inflammation or infection. Clinically, it may present with omphalitis, skin changes, or symptoms resembling those of an OMD fistula [3]. Definitive management requires surgical excision [3], as performed in our patient, and careful inspection for any additional OMD remnants is recommended during the procedure.

Preoperative imaging in this case illustrates a broader limitation in the diagnostic workup of complex OMD anomalies. Ultrasonography reliably characterized the superficial cystic component, correctly identifying its dimensions and tract toward the umbilicus, and is appropriately regarded as a first‐line modality given its accessibility and lack of radiation. However, it does not reliably characterize deeper or non‐cystic components such as thin fibrous bands or a discrete MD segment. Cross‐sectional modalities such as CT and MRI may offer improved delineation of tract anatomy and bowel wall involvement in some OMD presentations, but their sensitivity for identifying thin fibrous bands or small‐caliber MD remnants preoperatively remains limited, and such structures are frequently identified only intraoperatively regardless of the imaging modality used. In our patient, diagnostic laparoscopy was therefore pursued directly rather than pursuing further cross‐sectional imaging, allowing simultaneous full anatomical delineation and definitive surgical management under a single anesthetic, which also conferred economic benefit to the patient.

In our patient, a fibrous band connected the MD to the cystic OMD remnant, a phenomenon which was also observed by Ioannidis et al. [6], making the current case the second documented one in this aspect. Similar to other OMD fibrous bands, such connections are usually asymptomatic and often discovered only during surgery [3]. Although fibrous bands connecting the ileum to the umbilicus have been reported to cause bowel obstruction, as in Tahmasbi et al. [3], the band noted by Ioannidis et al. and in our report appeared to function only as a tether between the two omphalomesenteric duct remnants. This highlights the variability in presentation and anatomical configurations of such fibrous bands, further emphasizing the importance of careful intraoperative inspection for associated anomalies.

Histopathological diagnosis in this case was based on H&E staining alone, which demonstrated moderate nonspecific inflammation and hyperplasia of mucosal lymphoid tissue, with no evidence of granulomatous inflammation or malignancy. On morphological assessment, the reporting pathologist noted a lack of features suggestive of lymphoproliferative disease, mesenchymal malignancy (e.g., GIST), or epithelial malignancy, based on the H&E findings. Given this explicit morphological exclusion of the relevant differential diagnoses, additional immunohistochemical staining (e.g., CD45, cytokeratin, CD117/DOG1) was not pursued. We acknowledge that immunohistochemistry can offer further confirmatory confidence in select cases, and recommend its use where morphological findings are equivocal. In the present case; however, the H&E appearances were considered sufficiently reassuring by the reporting pathologist to exclude these differentials.

While OMD anomalies are seen in about 2% of individuals with their detection becoming increasingly uncommon as age advances [1, 3, 4], such cases have been widely reported. However, cases of more than two coexisting OMD anomalies in a single patient are quite rare. After an extensive literature review using relevant search terms across different databases, including PubMed, ScienceDirect, and Google Scholar, only three such studies were found. These include a 23‐year‐old male with an omphalomesenteric cyst drained through a sinus tract, fibrous ligament, and MD by Ioannidis et al. [6], an 8‐year‐old male with an umbilical polyp, MD, omphalomesenteric cyst and fibrous band by Nayak et al. [8], and a 12‐year‐old boy with a sinus tract connected to an omphalomesenteric cyst, which was further joined to an MD by another sinus tract by Jonuzi et al. [7]. These cases, together with the present one, are summarized in Table 1. While all these combinations are exceptionally rare and somewhat similar to the current case, the constellation of anomalies present in our 15‐year‐old female patient, namely an umbilical polyp, omphalomesenteric cyst joined by a sinus to the umbilicus, MD, and a fibrous band connecting the cyst to the MD, has, to the best of our knowledge, not been documented in the previous literature. Notably, our patient is the only female and the only adolescent among the four reported cases of multifocal OMD anomalies, in contrast to the male predominance and broader age range (8–23 years) seen in the other three reports (Table 1). This is a striking deviation given that symptomatic OMD remnants overall are significantly more common in males [1, 3], and reinforces the importance of maintaining a high index of suspicion for complex OMD pathology in adolescent females presenting with atypical umbilical complaints.

**TABLE 1:** Comparative summary of previously reported cases of coexisting omphalomesenteric duct anomalies, including the present case.

Author (Year)	Age	Gender	Specific combination of OMD anomalies	Surgical approach
Present case	15	Female	Umbilical polyp, omphalomesenteric cyst with secondary sinus to umbilicus, MD, and a fibrous band connecting the cyst to the MD.	Omphalectomy with complete excision of the umbilical polyp, sinus tract, and cyst; segmental ileal resection with end‐to‐end ileo‐ileal anastomosis.
Ioannidis et al. (2012) [6]	23	Male	Omphalomesenteric cyst drained through a sinus tract, fibrous ligament, and MD.	Semicircular infraumbilical incision with resection of the sinus tract, cyst, and fibrous band; Meckel's diverticulum removed by transverse wedge excision (no bowel resection/anastomosis).
Nayak et al. (2015) [8]	8	Male	Umbilical Polyp, MD, omphalomesenteric cyst, and fibrous band.	Laparotomy with resection of the involved intestinal segment, MD, cyst, and fibrous band.
Jonuzi et al. (2024) [7]	12	Male	Sinus tract connected to an omphalomesenteric cyst, which was further joined to an MD by another sinus tract.	Cyst/sinus excised from the umbilicus; partial ileal resection with single‐layer ileo‐ileal anastomosis.

Abbreviations: MD, Meckel's diverticulum; OMD, omphalomesenteric duct.

Surgical correction of such anomalies is generally the preferred approach. In our patient, omphalectomy along with cystectomy was required; therefore, a laparoscopic approach was not undertaken. An alternative option was laparoscopic wedge resection with cystectomy alone, preserving the umbilicus. However, this approach carried a risk of recurrent omphalitis. To prevent this potential complication, an omphalectomy was performed. Furthermore, a neo‐umbilicus can be reconstructed in a second stage after discussion with the patient and family, taking into account the patient's young age and female gender, in order to restore cosmesis.

## Author Contributions

**Tayyab Riaz:** conceptualization, investigation, resources, writing – review and editing. **Mohammad Abdullah Hameed:** writing – original draft, visualization, data curation, writing – review and editing. **Ritik Singh:** writing – review and editing, supervision, project administration, validation.

## Funding

The authors have nothing to report.

## Ethics Statement

All procedures followed have been performed in accordance with the ethical standards laid down in the 1964 Declaration of Helsinki and its subsequent amendments.

## Consent

Written informed consent was obtained from the patient and her parents/legal guardians for the publication of this case report and any accompanying images.

## Conflicts of Interest

The authors declare no conflicts of interest.

## Data Availability

Data sharing not applicable to this article as no datasets were generated or analyzed during the current study.
